# CMOS-Compatible ZrO_2_‑Based Film for Photoplethysmography Sensors Enabling Accurate and Sensitive Health Monitoring

**DOI:** 10.1021/acsami.5c22131

**Published:** 2025-12-30

**Authors:** Nuno Estrócio, Ampattu R. Jayakrishnan, Katarzyna Gwozdz, Adrian Kaim, Ji Soo Kim, Alexandre Silva, Veniero Lenzi, Paweł Noszczyk, Surya Nair, Mário A. C. Castro Pereira, Luís S. A. Marques, Robert L. Z. Hoye, Judith L. MacManus-Driscoll, José P. B. Silva

**Affiliations:** † Physics Center of Minho and Porto Universities (CF-UM-UP), University of Minho, Campus de Gualtar, 4710-057 Braga, Portugal; ‡ Laboratory of Physics for Materials and Emergent Technologies, LapMET, University of Minho, 4710-057 Braga, Portugal; § Department of Experimental Physics, 414290 Wroclaw University of Science and Technology, Wroclaw 50-370, Poland; ∥ Department of Materials Science and Metallurgy, 2152 University of Cambridge, 27 Charles Babbage Road, Cambridge CB3 OFS, U.K.; ⊥ Department of Building Engineering, Faculty of Civil Engineering, Wrocław University of Science and Technology, Wrocław 50-370, Poland; # Inorganic Chemistry Laboratory, Department of Chemistry, University of Oxford, South Parks Road, Oxford OX1 3QR, U.K.

**Keywords:** self-powered photodetector, ferroelectric binary oxides, ferro–pyro–phototronic effect, photoplethysmography, healthcare 4.0

## Abstract

Photoplethysmography (PPG) is a simple noninvasive technique for the detection of multiple cardiovascular parameters, such as heart rate, blood oxygen saturation (SpO_2_), systolic blood pressure, and diastolic blood pressure. However, the current commercial PPG technology is limited by several factors, including rigidity, bulkiness, high cost, high power consumption of ∼10’s mW, poor operational stability under ambient conditions, and susceptibility to motion artifacts. In this work, we overcome many of these limitations using a novel self-powered, miniaturized, low-cost, stable PPG sensor based on a simple CMOS-compatible fluorite-type ferro/pyroelectric Hf_*x*_Zr_1–*x*_O_2_ thin-film photodetector device. Our novel self-powered photodetector shows 26% higher responsivity and 23% improved sensitivity (perfusion index of 3.7%) for sensing blood volume changes in microvascular tissues compared to conventional PPGs, and a very high accuracy (*<*2% error) in the estimation of SpO_2_. The simple sensor has strong prospects for replacing current PPG sensors for health monitoring applications.

## Graphical abstract

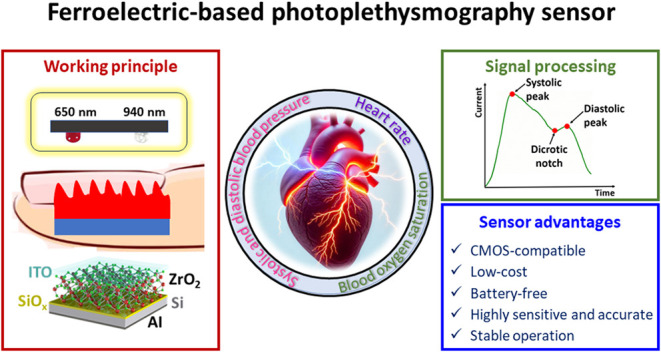

## 1 Introduction

The increased mortality rate per year due to cardiovascular diseases (CD) necessitates routine monitoring of vital signs such as heart rate (HR), peripheral capillary blood oxygen saturation (SpO_2_), and blood pressure (BP). Early detection of the variations in these vital signs can prevent 90% of CDs and thereby reduce the mortality rate.^1^

Photoplethysmography (PPG) is a low-cost, noninvasive technique that uses a simple circuit with a photodetector (PD) as a sensor and near-infrared (NIR) light source to track HR signals, and a NIR and red/green light source to estimate blood oxygen saturation (SpO_2_) in a pulse oximeter.^2^ However, the majority of PPG sensors use inorganic Si photodiodes for tracking health conditions.^2,3^ However, problems like rigidity, bulky nature, high expense associated with scalable fabrication,^4^ low operating temperature (≤20 °C), and the need for an external power source limit the application of semiconductor-based PPG devices.^2,5,6^ Despite advancements in sensor design and signal processing algorithms, current commercial PDs consume tens of milliwatts of power to detect PPG signals.^7^ In addition, factors like low perfusion index (0.1–3%), insufficient operational stability under ambient conditions, motion artifacts, and semiconductor noise significantly affect the performance parameters of PPG sensors, such as the signal-to-noise ratio (SNR), dynamic range (DR), and signal quality.^7,8^ In addition, to reduce heat production and eliminate the requirement for a large inorganic battery, which restricts the size and form factor of PPG sensors, self-powered photodetectors (SPDs) must be developed as sensors to overcome the above-mentioned problems.^7,8^

For example, organic SPDs are currently being investigated as PPG sensors. However, the reaction of constituent organic molecules in the PDs with water and oxygen molecules in the air, particularly at the electrode interface, significantly affects the PPG signal quality. In this sense, inorganic PPG sensors are preferred due to their higher stability.^8^ Therefore, an eco-friendly inorganic SPD with a simple structure, good crystallinity, high stability, and room-temperature functionality shows new possibilities in PPG devices for medical diagnosis.

Beyond the enormous potential for information and energy storage,^9–11^ ferroelectric (FE) fluorite HfO_2_- and ZrO_2_-based oxides have started to attract attention in sensing devices.^12–16^ However, none of these works have demonstrated the potential of these materials in PPG sensors. Therefore, in this work, we demonstrate for the first time the possibility of a self-powered, simple-structured, CMOS-compatible inorganic-based SPD as a sensor in a PPG device with high accuracy in SpO_2_ measurement (<2%) due to the high SNR value (41 dB) and high sensitivity due to the high PI value (3.7%), which is 23% higher than that found in commercial PPG sensors. This excellent performance was achieved by exploiting the ferroelectric, pyroelectric, and photovoltaic functionalities of FE Hf_*x*_Zr_*1‑x*_O_2_ (HZO, *x* = 0, 0.30, 0.50) thin films. Besides monitoring the HR, the ZrO_2_ sensors show the capability of monitoring systolic and diastolic blood pressure, revealing their potential for use as a battery-free PPG device.

## 2 Results and Discussion

A Si/SiO_*x*_/HZO structure was fabricated by depositing 5 nm-thick Hf_*x*_Zr_1–*x*_O_2_ (HZO, with *x* = 0, 0.30, and 0.50) by ion-beam sputtering (IBS) onto p-type (100) Si/2.5 nm SiO_*x*_ (Si-Mat). All details regarding the growth and characterization of the devices are provided in Section 4.

Figure 1a shows the GIXRD patterns of the HZO (50/50), HZO (30/70), and ZrO_2_ films deposited on Si/SiO_*x*_ substrates. The diffraction peak at 2θ ∼30° confirms the presence of the (111) planes of the *o-*phase in all films.^15,17^ In addition, a peak shift of the (111) peak toward higher angles was observed, which was attributed to the higher concentration of Zr in HZO.^14^ The presence of small peaks near 2θ ∼28.4° and 2θ ∼31.6° for the HZO 50/50 and HZO 30/70 films suggests the presence of a secondary monoclinic phase, with peaks assigned to the (1̅11) and (111) planes, respectively.^18,19^ The phase content in the different films is discussed in the SI (Figure S1 and Table S1).

**Figure 1:**
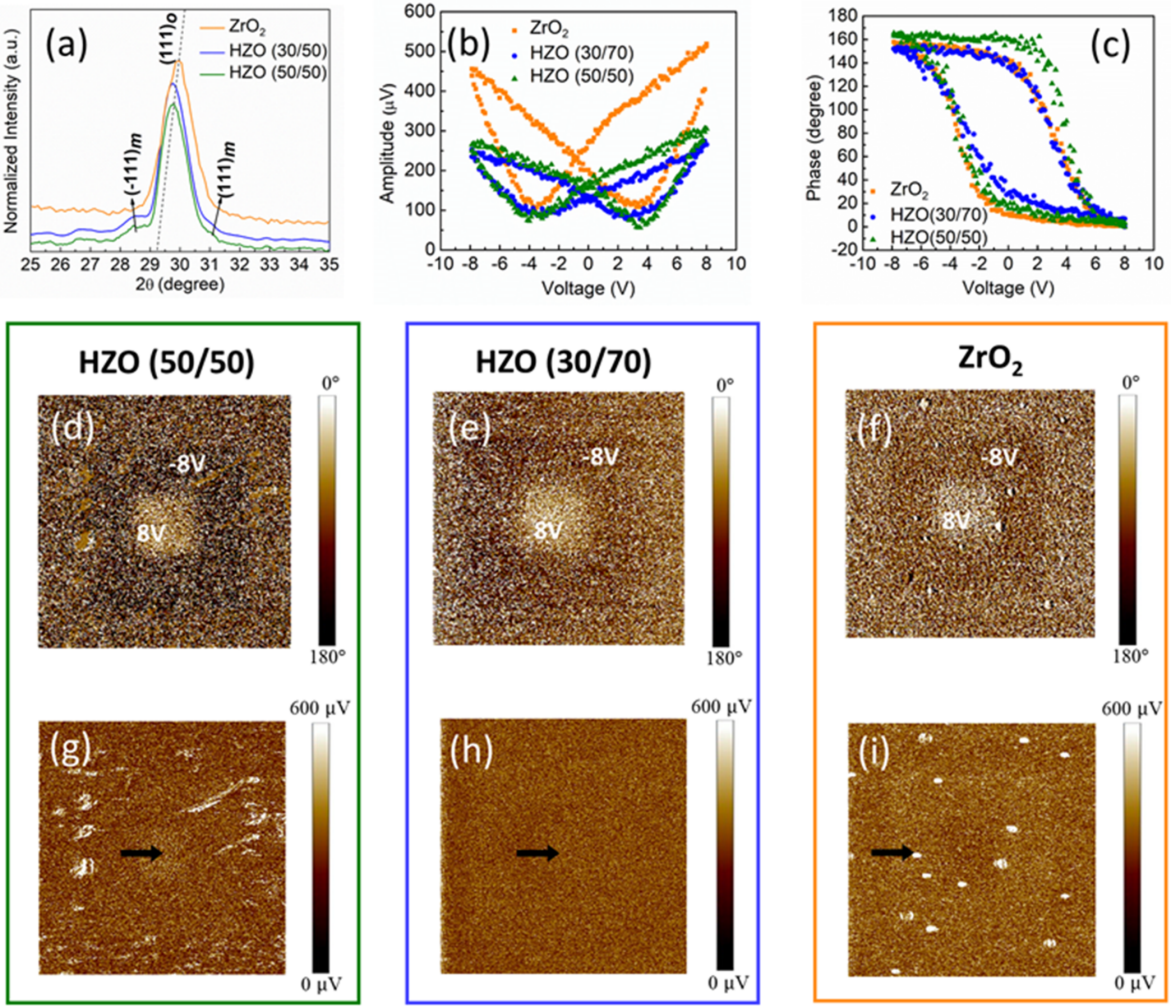
(a) X-ray diffraction pattern of the HZO films. (b, c) Piezoresponse force spectroscopy (PFS) out-of-plane hysteresis loops in phase and amplitude with *V*_ac_ of 1 V for HZO (50/50) and *V*_ac_ of 2 V for HZO (30/70) and ZrO_2_ at 10 kHz. (d–f) Vertical PFM (VPFM), out-of-plane polarization response of the phase for HZO (50/50), HZO (30/70), and ZrO_2_ postpolarized with −8 and 8 V. (g–i) VPFM response of the amplitude for HZO (50:50), HZO (30/70), and ZrO_2_ postpolarized with −8 and 8 V. The black arrows indicate the region where the out-of-plane amplitude at the boundary of the oppositely polarized regions was investigated, as shown in Figure S2.

Figure 1b,c presents the PFM and piezoresponse force spectroscopy (PFS) results of the HZO films. The out-of-plane phase and amplitude responses of the HZO films obtained using the PFS are shown in Figure 1b,c. The phase and amplitude indicate the presence of piezo/ferroelectricity in the HZO (50/50), HZO (30/70), and ZrO_2_ films.^20,21^ Here, a *V*_dc_ of ±8 V was used for writing, while for reading, a *V*_ac_ of 1 V for HZO (50/50) and 2 V for HZO (30/70) and ZrO_2_, respectively, at 10 kHz. The coercive voltages were extracted and averaged from the minima of amplitude loops. The coercive voltages for HZO (50/50), HZO (30/70), and ZrO_2_ are 3.85 V, 3.25, and 3.15 V, respectively, although phase saturation occurs at ∼6 V for all the samples. Additionally, spatial polarization was performed using a *V*_dc_ of ±8 V with a *V*_ac_ of 1 V for HZO (50/50) and 2 V for HZO (30/70) and ZrO_2_, with a scan size of 5 μm. The vertical PFM (VPFM) images with oppositely polarized states are shown in Figure 1d–i, with a constant level of phase and amplitude across the polarized region, which clearly reveals reversible polarization states.^20–23^ Also, based on the contrast from the spatial polarization, the pristine state is multidomain with a mixture of randomly oriented domains. In addition, for HZO­(50/50), HZO­(30/70), and ZrO_2_, the presence of a domain wall is confirmed, as shown in Figure S2a–c. A sharp dip at the boundary of the oppositely polarized regions was observed, as marked with black arrows in Figure 1g–i. Thus, PFM confirms the ferroelectric/piezoelectric response in our HZO thin films.^20–23^

Figure 2a–c shows the *I–t* response of an Al/Si/SiO_*x*_/HZO/ITO device under a zero-bias voltage, illuminated with an LED light of wavelength 940 nm at a fixed power density of 1.4 mW/cm^2^ and a pulse repetition rate of 10 Hz. The transient responses of the Al/Si/SiO_*x*_/HZO/ITO devices clearly reveal a photoresponse under the LED on and off states. This is achieved by the coupling of ferroelectric, pyroelectric, and photovoltaic effects, also known as ferro*–*pyro*–*phototronic effect.^15,16^ It is important to mention that ZrO_2_ and Hf_*x*_Zr_1–*x*_O_2_ layers do not absorb the incident light. Instead, the instant illumination causes a slight surface heating, as ITO partially absorbs the light (see the absorbance spectrum versus wavelength for an ITO thin film deposited on a glass substrate in Figure S3), which results in a change in the effective polarization and, consequently, the surface potential, and the so-called pyro*–*phototronic potential, leading to the appearance of a sharp peak.^12,16,24,25^

**Figure 2:**
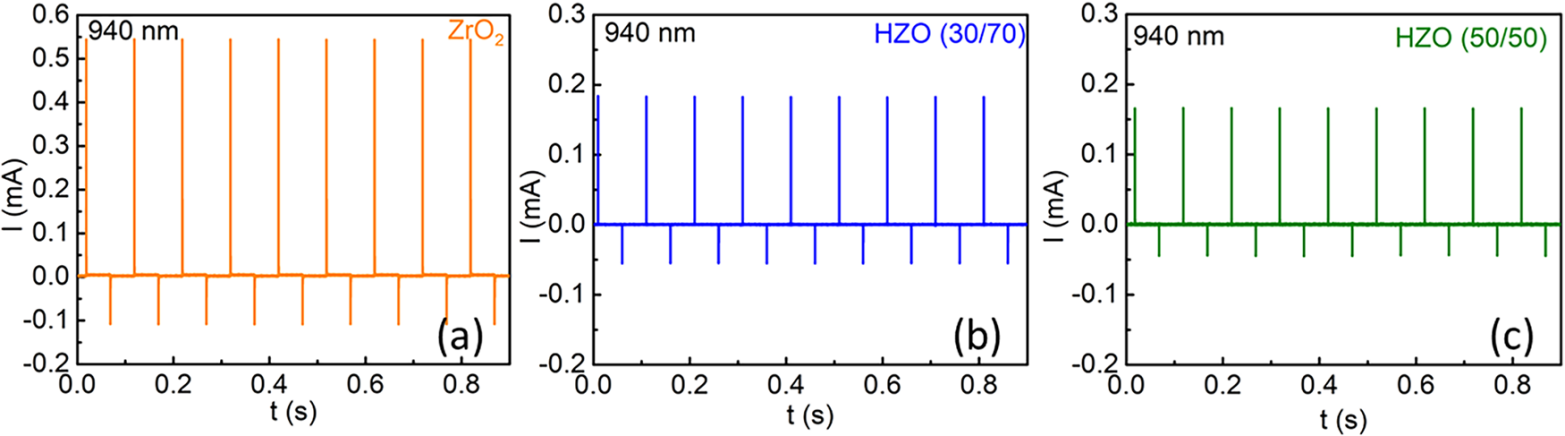
(a–c) *I–t* curves for the Al/Si/SiO_*x*_/HZO/ITO devices measured for light of 940 nm wavelength at a fixed chopper frequency of 10 Hz and a fixed power density of 1.4 mW/cm^2^ at 0 V.

The important figure-of-merits determining the photodetection performance of a PD such as responsivity (*R*), detectivity (D*), and sensitivity (*S*) are described in SI. The calculated values of *R*, D*, and *S*, as well as the rise time (τ_r_) and fall time (τ_f_), as a function of the HZO composition, are shown in Figure 3a,b, respectively. Notably, all HZO devices showed a very good photoresponse with an ultrafast response time under zero-bias voltage. In particular, the Al/Si/SiO_*x*_/ZrO_2_/ITO SPD showed optimum performance in terms of *R*, D*, *S*, τ_r_, and τ_f_.

**Figure 3:**
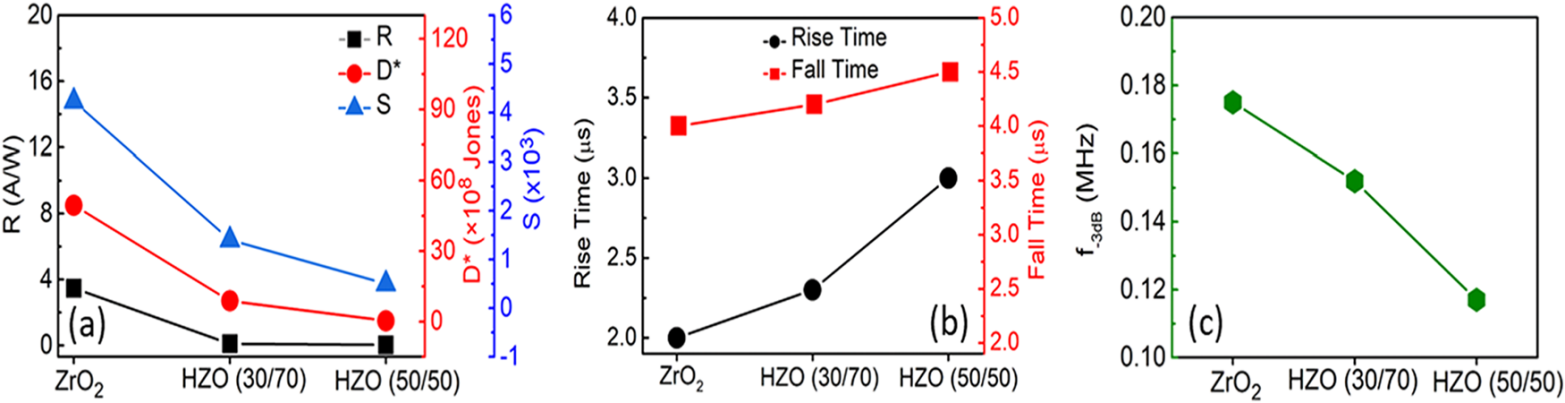
(a) Responsivity (*R*), detectivity (D*), and sensitivity (*S*) as functions of composition. (b) Rise and fall times and (c) cutoff frequency (*f*_–3 dB_) as a function of composition.

In addition, the cutoff frequency (*f*_–3 dB_) was calculated as detailed in SI and is shown in Figure 3c as a function of the HZO composition. In general, the bandwidth required for commercial sensing applications is 0.1 MHz,^26,27^ which is below the value obtained for the present PDs.

While it is well-known that a high ferroelectric polarization is achieved in HZO (50/50) films (∼30 μC/cm^2^) when compared to ZrO_2_ films (∼9.3 μC/cm^2^), the pyroelectric response has been much less investigated^10,28,29^ and there are no studies on the effect of the composition on the pyroelectric properties of HZO (30/70 and 50/50) and ZrO_2_. In addition, it was recently shown that the pyroelectric coefficient in HfO_2_-based films increases with decreasing thickness.^14,28^ Therefore, we kept the thickness of the films as low as possible in order to obtain a pronounced ferro*–*pyro*–*phototronic effect and therefore a better photodetector performance. A further decrease in the thickness will cause a high leakage current, which will degrade the photodetector performance. To further elucidate the effect of composition on the pyroelectric response of SPDs, ab initio molecular dynamics simulations were performed to calculate the pyroelectric coefficient at 300 K for HZO (30/70 and 50/50) and ZrO_2_ (Figure 4a). It is possible to observe that the pyroelectric coefficient for HZO (50/50) is almost twice that of ZrO_2_.^30^ Therefore, one should expect an enhanced PD performance for HZO (50/50) due to improved ferroelectric and pyroelectric performance.

**Figure 4:**
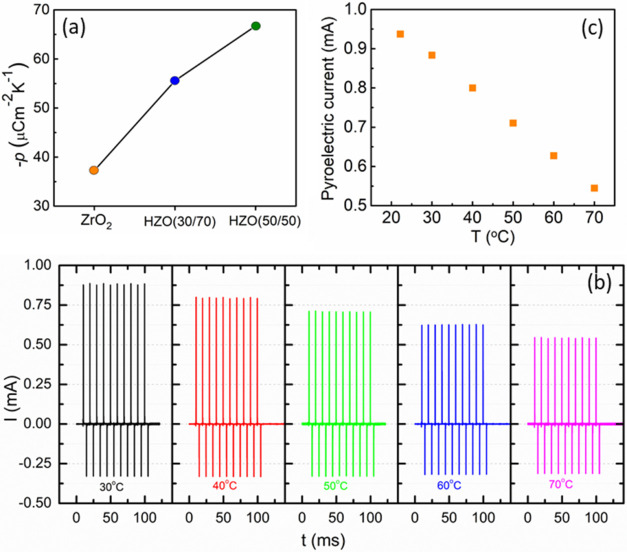
(a) Ab initio molecular dynamics calculation of the pyroelectric coefficient as a function of the Zr content in Hf_*x*_Zr_1–*x*_O_2_. The line is a guide for the eye. (b) Temperature dependence of the *I–t* curves for the Al/Si/SiO_*x*_/ZrO_2_/ITO devices measured for a 650 nm wavelength light at a fixed chopper frequency of 100 Hz and a fixed power density of 251 mW/cm^2^ at 0 V. (c) Temperature dependence of the maximum pyroelectric current.

However, the unexpectedly lower photoresponse performance of the HZO (50/50) and HZO (30/70)-based PDs might be ascribed to the fact that a pure *o-*phase film is not achieved in the HZO films, as in ZrO_2_ films.

In PPGs, red light is usually used since it can be easily transmitted by the blood vessel and is useful to measure physiological parameters such as SpO_2_.^31,32^ To confirm the sensitivity of our devices to red light, the photoresponse of a device with the optimum composition film, Hf_*x*_Zr_1–*x*_O_2_, with *x* = 0, i.e., ZrO_2_, Al/Si/SiO_*x*_/ZrO_2_/ITO, was studied. Thus, the SPD was measured under illumination at a 650 nm wavelength with a fixed frequency of 10 Hz and a power density of 540 mW/cm^2^ (Figure S4a). The device showed a very good photoresponse in terms of *R* (159 mA/W), D* (4.5 × 10^6^ Jones), *S* (6.9 × 10^3^), τ_r_ (3.5 μs), and τ_f_ (3.2 μs), under red light (Figure S4b). In order to confirm the ferro*–*pyro*–*phototronic effect, thermal imaging was performed in the dark (Figure S4c) and under 180 s of light illumination at a 650 nm wavelength with a power density of 540 mW/cm^2^ (Figure S4d) for the Al/Si/SiO_*x*_/ZrO_2_/ITO device. It is possible to observe that the temperature change (Δ*T*) between a dark condition and 650 nm irradiation was significant, and the surface of the device heats up by around 3.4 °C. Figure S5 shows the signal of the Al/Si/SiO_*x*_/ZrO_2_/ITO device under laser illumination with a wavelength of 650 nm, power density of 251 mW/cm^2^, and chopper frequency of 100 Hz. In Figure S5a, the testing conditions correspond to positive prepoling, whereas in Figure S5b, the testing conditions are reversed. It can be observed that the direction of the current is reversed in the second case, but the magnitude of the current remains unchanged.

For comparison, an HZO-free reference Al/Si/SiO_*x*_/ITO device was fabricated and tested. No photoresponse was observed, indicating that HZO is crucial for sensing (Figure S6a). Moreover, the existence of the SiO_*x*_ native layer is relevant as a passivation layer to reduce the leakage current. Figure 4b shows the temperature dependence of the *I*–*t* curves for the Al/Si/SiO_*x*_/ZrO_2_/ITO devices measured for light of 650 nm wavelength at a fixed chopper frequency of 100 Hz and a fixed power density of 251 mW/cm^2^, at 0 V. From Figure 4c, it can be concluded that the maximum pyroelectric current decreases linearly with temperature. In addition, the zero in the pyroelectric current can be extrapolated to be at a temperature of 136 °C, which is in agreement with the phase transition temperature in ferroelectric ZrO_2_.^31^

The pyroelectric coefficient (*p*) was calculated using eq 1:^32,33^

1 $$p=\frac{I_{p}}{A}\cdot \frac{1}{\frac{dT}{dt}}$$

where *I*_p_ is the pyroelectric current, *A* is the electrode area, and d*T*/d*t* is the temperature ramp rate. By considering the *I*–*t* curves measured at two different temperatures (e.g., 30 and 40 °C) and the additional time it takes to reach the same current in the case of the experiment performed at a higher temperature, *p* was estimated to be 18.2 μC m^–2^ K^–1^ at 30 °C. This value is of the same order of magnitude as the calculated one and is in good agreement with the measured value for 45 nm-thick ZrO_2_ films, using the Sharp–Garn method.^29^

Figure 5a shows a simple structured PPG sensor that incorporates a light source and Al/Si/SiO_*x*_/ZrO_2_/ITO SPD to test the noninvasive cardiac cycle (HR monitoring) and the SpO_2_ in a human body.

**Figure 5:**
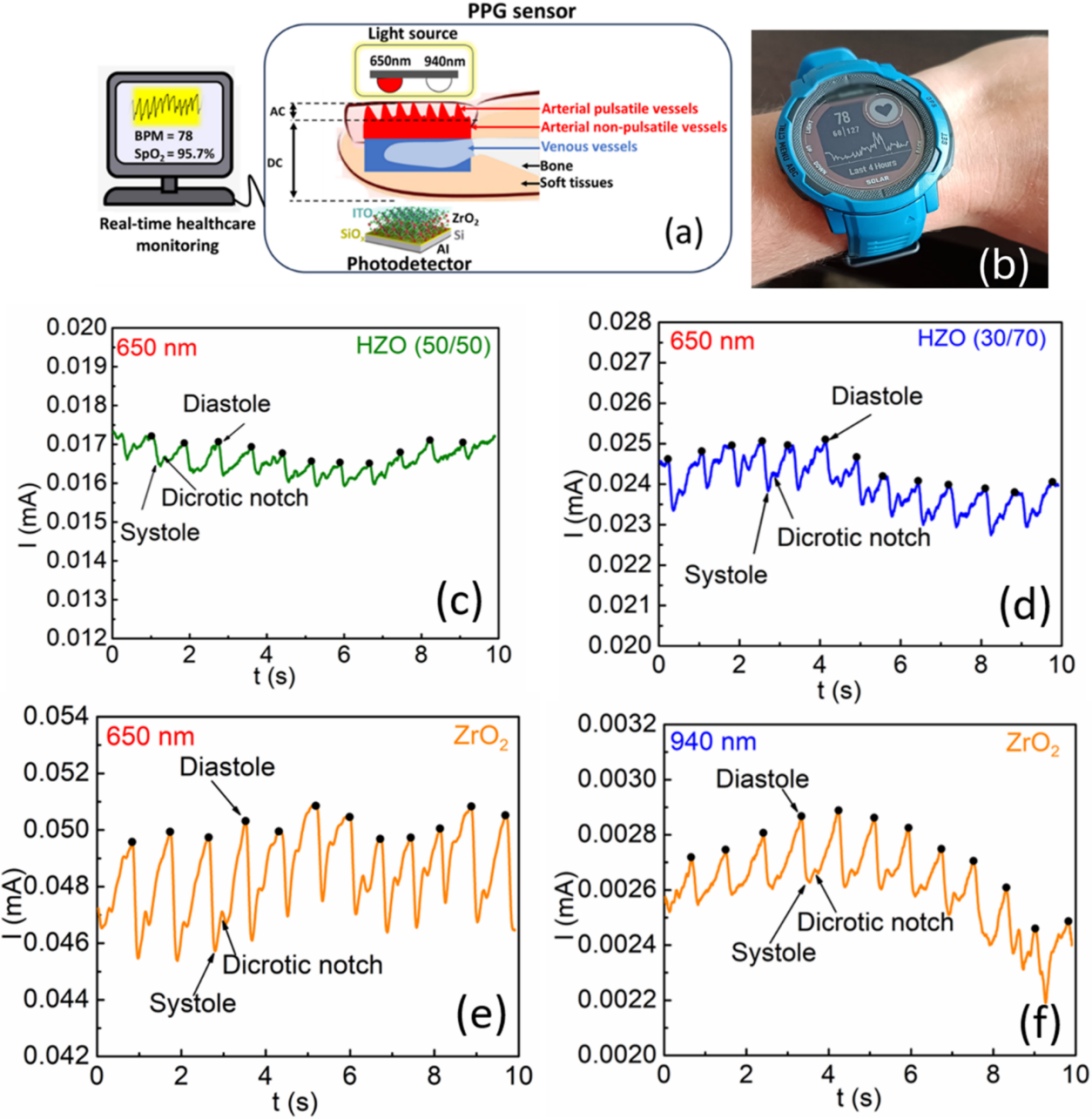
(a) Schematic representation of the PPG using the Al/Si/SiO_*x*_/ZrO_2_/ITO device used in this work. Here, the AC component corresponds to the light absorption ascribed to the variation in the diameters of pulsatile vessels (arterial pulsatile vessels), and the DC component corresponds to the light absorption at places such as the arterial nonpulsatile vessels, venous vessels, bone, and soft tissues. Here, BPM refers to beats per minute. (b) HR was measured using a commercial Garmin wristwatch. Pulse signal measured using (c–e) the Al/Si/SiO_*x*_/HZO/ITO device at 650 nm and (f) the Al/Si/SiO_*x*_/ ZrO_2_/ITO device at 940 nm under zero-bias voltage.

Typically, a PPG sensor uses NIR and visible (blue, red, or green) light. Red and NIR light (800–950 nm) reach deeper vascular tissues and enable SpO_2_ to be measured more accurately. The red light wavelength range is also less affected by the skin tone than blue and green, and is more often used in medical-grade equipment.^34^ PPG uses the change in the intensity of the reflected and transmitted light from the red and NIR signals in the blood flow volume (volume change of blood vessels in the systole and diastole phase upon each cardiac cycle) in the microvascular tissue bed arising from changes in strain on the finger. In addition, Figure 5a shows that the signal is composed of an AC component, which is the pulsatile signal, and a DC component, which is the nonpulsatile signal. This variation in the electrical signal converted by the PPG device is used to evaluate HR and SpO_2_.^35,36^ The PPG signal profile shown in Figure 5c–e gives the *I–t* response from different Al/Si/SiO_*x*_/HZO/ITO SPDs when the finger of a volunteer was placed between the red light, using a laser pulse of 500 Hz for 10 s, and the SPDs. A detailed description of the experimental procedure used to analyze the results is shown in Figure S6b,c. In addition, a stable response is observed for up to 5000 light ON/OFF cycles. In all cases, it was possible to identify the systole and diastole from the blood flow in humans, together with 12 cardiac cycles within 10 s. The HR was estimated to be 78 beats per minute for all the devices. The HR value obtained using our SPD device is similar to that obtained with a commercial Garmin wristwatch, as shown in Figure 5b,^37^ demonstrating the high accuracy of our Al/Si/SiO_*x*_/HZO/ITO SPDs. However, by comparing Figure 5c–e, it is possible to conclude that the magnitude of the AC component is at least 2 times higher for the Al/Si/SiO_*x*_/ZrO_2_/ITO SPDs, which indicates their higher sensitivity for detecting blood volume changes in the microvascular bed of tissue.

Since the PPG signals obtained exhibited clear waveforms, with the aim of estimating SpO_2_, we measured the *I–t* response of the Al/Si/SiO_*x*_/ZrO_2_/ITO SPD when it was exposed to NIR light using LED pulses with a repetition rate of 500 Hz for 10 s, as shown in Figure 5f. We extracted the peak values of the PPG waveforms and determined the ratio (*R*_OS_) of pulsatile (AC) to nonpulsatile (DC) signals at the two wavelengths (red and NIR light) by using eq 2 as follows:^35,36^

2 $$R_{\mathrm{OS}}=\frac{AC_{\mathrm{red}}/DC_{\mathrm{red}}}{AC_{\mathrm{NIR}}/DC_{\mathrm{NIR}}}$$

where *R*_OS_ represents the ratio of the AC (pulsatile) and DC (nonpulsatile) components corresponding to the diffusion of light through the arterial blood, as shown in Figure 4a.^35,36^

The *R*_OS_ value for the Al/Si/SiO_*x*_/ZrO_2_/ITO SPD was estimated to be 0.57. The SpO_2_ was calculated using eq 3:^35^

3 $$\mathrm{Sp}O_{2}\%=110-R_{\mathrm{OS}}\times 25$$

The value of SpO_2_ was found to be 95.7%.

The perfusion index (PI) indicates the strength of the pulse signal detected by a PPG sensor and was estimated using eq 4:^7,37^

4 $$\mathrm{PI}=\frac{\mathrm{AC}}{\mathrm{DC}}$$

where AC is the pulsatile signal, and DC is the nonpulsatile signal measured with NIR light. The PI for the Al/Si/SiO_*x*_/ZrO_2_/ITO SPD was found to be 3.7%, which is higher than that obtained with other PPG sensors that typically range from 0.1% to 3%.^38^ Therefore, the Al/Si/SiO_*x*_/ZrO_2_/ITO SPD exhibits 23% higher PI when compared to commercial PPG sensors.^39,40^ This important feature allows avoiding the potential use of a dc photocurrent rejection method to decrease the large unwanted dc component, as it is used with PPG sensors based on Vishay VEMD 6060 × 01 silicon PIN photodiodes.^38^ Therefore, the PI value confirms the strong sensitivity of the Al/Si/SiO_*x*_/ZrO_2_/ITO SPD. Furthermore, the signal-to-noise ratio (SNR) was calculated using eq 5:^41,42^

5 $$\mathrm{SNR}=20\;\log_{e}\left(\frac{A_{\mathrm{Signal}}}{A_{\mathrm{Noise}}}\right)\;\left(\mathrm{dB}\right)$$

where the SNR was evaluated from the raw waveform using the signal and noise.^41^ *A*_Signal_ is the amplitude of the PPG signal, and *A*_Noise_ is the amplitude of noise.^41,42^ The calculated SNR value was found to be 41 dB. The SNR value required to measure SpO_2_ with an error of <2% is above 39 dB.^7^ Hence, our SPD device successfully monitors real-time SpO_2_ with high accuracy. Furthermore, the systolic blood pressure (SBP) and diastolic blood pressure (DBP) were estimated using eqs 6 and 7:^35^

6 SBP=−24.6×h+25.7×S+110.5

7 DBP=27.8×h−30.87×S+83.34

where *h* represents the height ratio of the diastolic peak (*h*_2_) to the systolic peak (*h*_1_), and *S* represents the proportion of the systolic phase (*S*_ABCJ_/*S*_AFGJ_). A schematic representation of the typical PPG waveform for the calculation of blood pressure (SBP and DBP) is shown in Figure S7. The obtained values of SBP and DBP are in the ranges 114–116 mmHg and 75–77 mmHg, respectively. This is in good agreement with the normal values of SBP (<120 mmHg) and DBP (<80 mmHg) reported for adults.^43^ Thus, the Al/Si/SiO_*x*_/ZrO_2_/ITO SPD can accurately monitor and extract various physiological information.

Moreover, the current FE ZrO_2_-based SPD operating at room temperature means that this simple ferroelectric device rivals commercial Si-based PDs because Si-based PDs require an external power source and low operational temperature for their smooth functioning,^1,2^ whereas the FE ZrO_2_-based SPDs do not. In addition, these materials can also be integrated into flexible wearable devices, showing very stable operation up to 10^7^ cycles,^44–46^ demonstrating the potential of SPDs for flexible healthcare monitoring.

Therefore, the real-time response of the current PPG devices offers significant advantages over commercial oximeters and PPG sensors in wristwatches, as well as over other devices that are currently being reported in the literature, with a performance benchmark in Table S2.

## 3 Conclusions

This work successfully demonstrates a simple, cost-effective, high-performance, and CMOS-compatible photoplethysmography sensor for healthcare monitoring. The working principle of the Al/Si/SiO_*x*_/ZrO_2_/ITO self-powered photodetector is based on the triple combination of the ferroelectric, pyroelectric, and photovoltaic properties. Compared to commercial photoplethysmography sensors, the current devices exhibit 26% higher responsivity and 23% improved sensitivity. A perfusion index to near-infrared light of 3.7%, together with a signal-to-noise ratio of 41 dB, confirmed the high sensitivity and accuracy with an error of <2% in the SpO_2_ measurement.

Overall, our work demonstrates the very high potential of self-powered ferroelectric ZrO_2_-based photodetectors for use in photoplethysmography sensors, in future Healthcare 4.0 applications.

## 4 Materials and Methods

A Si/SiO_*x*_/HZO structure was formed by depositing 5 nm-thick Hf_*x*_Zr_1–*x*_O_2_ (HZO, with *x* = 0, 0.30, and 0.50), namely, ZrO_2_, HZO (30/70), and HZO (50/50) films by ion-beam sputtering (IBS) onto p-type (100) Si/2.5 nm SiO_*x*_ (Si-Mat). The growth and annealing conditions for the deposition of HZO films are given in our previous work.^14^ Al/Si/SiO_*x*_/HZO/ITO devices were then made by depositing top ITO and bottom Al electrodes, as reported in previous work.^15^

The structural characterization of the HZO layers was performed using grazing incidence X-ray diffraction (GIXRD), which was carried out in a high-resolution Panalytical Empyrean vertical diffractometer with an incident angle of 1.0°, a 1/2° incident divergence slit, and a receiving slit opening of 1.10 mm. The integration time was 10 s for each step. Piezoresponse force microscopy (PFM) was performed using a Bruker Multimode 8 atomic force microscope with platinum (Pt)-coated NSC35 tips (MikroMasch) with a spring constant of 5.4 N/m. The sample was mounted on a metallic disc, where V_dc_ was applied via the bottom p-Si while grounding the Pt PFM tip. The PFM response was detected with a V_ac_ (varying between samples) of 2 V at 10 kHz. For testing the NIR-sensing capabilities, the *I*–*t* curves were measured using a current amplifier (Keithley 428) and the National Instrument I/O card (PCI-6251) programmed in LabVIEW. The Al/Si/SiO_*x*_/HZO/ITO devices were illuminated using a light-emitting diode (LED) with a wavelength of 940 nm and a laser with a wavelength of 650 nm. The power was measured with a Nova II by Ophir. The light was digitally chopped, with a pulse repetition rate fixed at 10 Hz for the SPD tests, while a 500 Hz pulse repetition rate was used for PPG sensors. The volunteer provided informed consent for participation in the experiment and for the publication of the results in written form. Prior to these tests, the devices were positively prepoled in order to maximize the PD performance utilizing the ferro–pyro–phototronic effect, as described in ref 2. For the temperature-dependent *I*–*t* measurements, the temperature of the substrate was stabilized by using a Peltier device and a custom-built, computer-controlled PID driver. IR thermal images were recorded in the dark and under 650 nm light illumination using a thermal imaging camera FLIR T640.

Ab initio molecular dynamics (AIMD) simulations were performed with VASP, version 6.4.3.^47–49^ The GGA approximation was adopted using the PBESol functional.^50^ The orbitals 4s, 4p, 5s, 4d for Zr and 5s, 5p, 6s, 5d for Hf were treated explicitly. 96-atoms *o*-phase cells with the polarization axis aligned with the (001) direction were built for the Hf_*x*_Zr_1–*x*_O_2_ systems, where for *x* = 0, 0.3, and 0.5 special quasi-random structures (SQS) were considered.^51^ A plane wave energy cutoff of 600 eV was used in all calculations, with a 2 × 2 × 2 γ-centered K-point grid. To implement the NPT ensemble, the Parrinello–Rahman method was used in conjunction with a Langevin thermostat and barostat.^52,53^ A time step of 1 fs was used. After an equilibration step of 1 ps, data production runs of 3 ps were run at the desired temperature and pressure conditions. The temperature-dependent spontaneous polarization along the *c-*axis *P*_s_ was calculated on the ensemble-averaged structures using the Berry phase approach.^54–56^ The pyroelectric coefficient *p* at 300 K was calculated using $p=\frac{\partial P_{s}\left(T\right)}{\partial T}$ , namely *P*_s_ was obtained at 200 K, 300 K and 400 K and then the derivative was calculated as a central difference.^57^ The obtained values were scaled by a factor of 1/√3 to take into account the different orientations of the experimental films (111-oriented) compared to the simulated bulk (001-oriented).

## Supplementary Material



## Data Availability

The data will be made available upon request.

## Notes

Further details on the phase content and PFM analysis of the different samples; absorbance spectrum versus wavelength for an ITO thin film deposited on a glass substrate; detailed photodetector performance evaluation; detailed photoplethysmography measurements and performance benchmark (PDF)
