# Supplementary material for: CMOS-Compatible ZrO2‑Based Film for Photoplethysmography Sensors Enabling Accurate and Sensitive Health Monitoring
Source: ACS Appl Mater Interfaces. 2025 Dec 30;18(1):2130–8. doi: 10.1021/acsami.5c22131 (PMC12818714; doi:10.1021/acsami.5c22131)
Supplement: Supplementary file 1 [file am5c22131_si_001.pdf]

Supporting Information

CMOS compatible ZrO_2–based film for photoplethysmography sensors enabling accurate and sensitive health monitoring

Nuno Estrócio[1,2,δ], Ampattu R. Jayakrishnan,[1,2,δ], Katarzyna Gwozdz[3], Adrian Kaim[3], Ji Soo Kim,[4] Alexandre Silva[1,2], Veniero Lenzi[1,2], Paweł Noszczyk[5], Surya Nair[1,2], Mário A. C. Castro Pereira[1,2], Luís S. A. Marques[1,2], Robert L. Z. Hoye[6], Judith L. MacManus-Driscoll[4*], José P. B. Silva[1,2,*]

[1]Physics Center of Minho and Porto Universities (CF-UM-UP), University of Minho, Campus de Gualtar, 4710-057 Braga, Portugal

[2]Laboratory of Physics for Materials and Emergent Technologies, LapMET, University of Minho, 4710-057 Braga, Portugal

[3]Department of Experimental Physics, Wroclaw University of Science and Technology, Wroclaw 50-370, Poland

[4]Dept. of Materials Science and Metallurgy, University of Cambridge, 27 Charles Babbage Rd., Cambridge, CB3 OFS, U.K.

[5]Department of Building Engineering, Faculty of Civil Engineering, Wrocław University of Science and Technology, Wrocław, 50-370, Poland

[6]Inorganic Chemistry Laboratory, Department of Chemistry, University of Oxford, South Parks Road, Oxford, OX1 3QR, UK

[δ] These authors contributed equally to this work.

[*] Corresponding authors' e-mails: josesilva@fisica.uminho.pt and jld35@cam.ac.uk

Phase content analysis

In order to confirm the phase content in the HZO (50/50), HZO (30/70), and ZrO_2 films, we deconvoluted the XRD peaks (Fig. S1(a-c)). The fitting clearly reveals the presence of the monoclinic (*m*)-phase peaks in the HZO (50/50) and HZO (30/70) films[1,2]. To evaluate the evolution of the *m*-phase content with the composition in the HZO (50/50) and HZO (30/70) films, we calculated the ratio between the area of the *m*-($\bar{1}11$) peak to the *o*-(111) one. As demonstrated in Table S1, this ratio increases with Hf content in HZO[3]. Usually, a higher crystallization temperature is needed for the HZO films containing more Hf (178.49 amu) relative to Zr (91.224 amu), since heavier atoms will require more thermal energy to diffuse and crystallize[3]. For this reason, the lower crystallization temperature of thin ZrO_2 films results in better back-end of line compatibility[4].

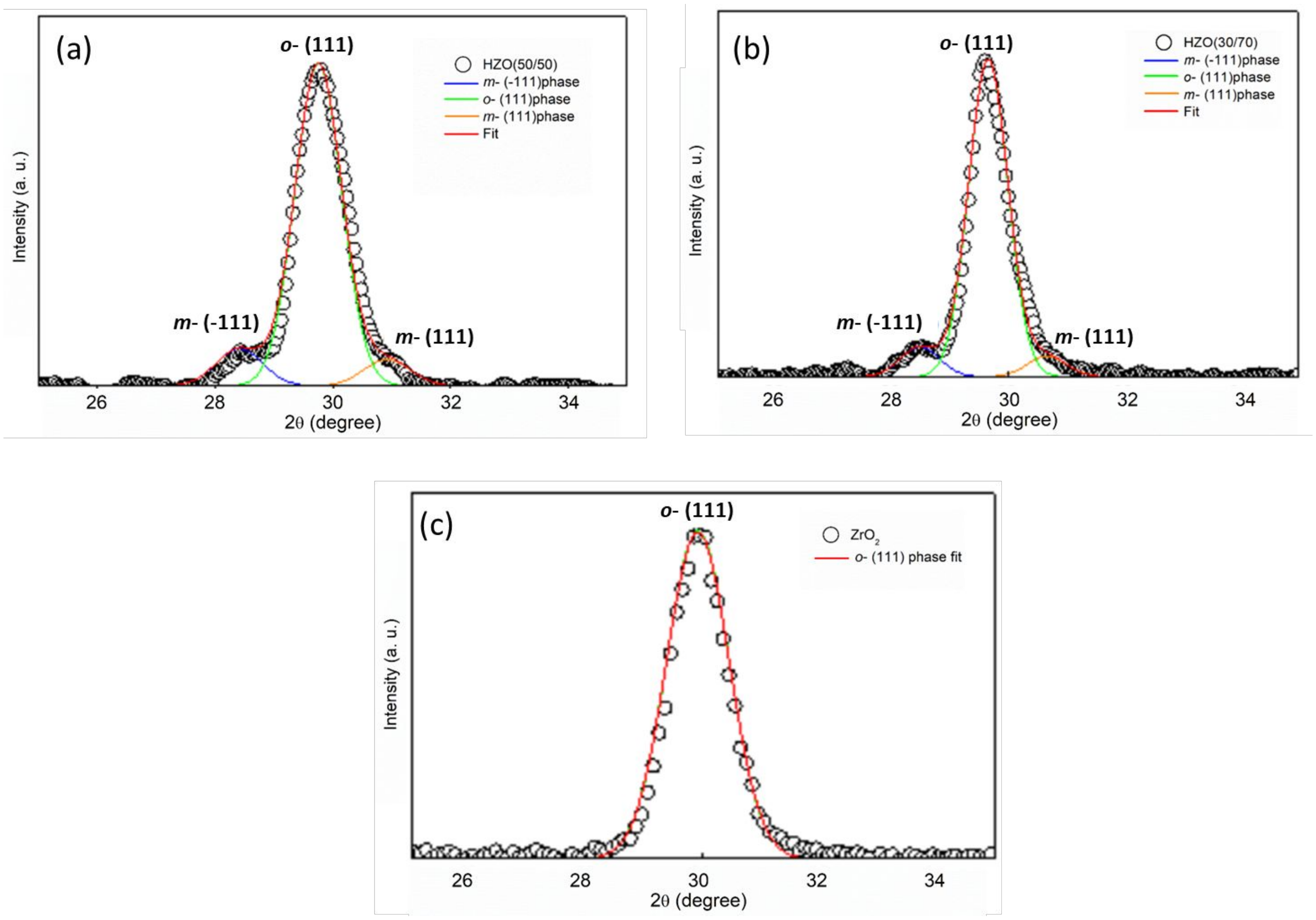

Figure S1. Deconvoluted GIXRD pattern of (a) HZO (50/50), (b) HZO (50/50), and (c) ZrO_2.

Table S1: Ratio between the area of the *m*-(-111) peak to the *o*-(111) one for the different HZO films grown on Si/SiO_x substrate.

**Thin film composition**	***m*- (-111) phase/*o*-(111) phase area ratio**
HZO (50/50)	0.11
HZO (30/70)	0.08
ZrO_2	0

PFM analysis

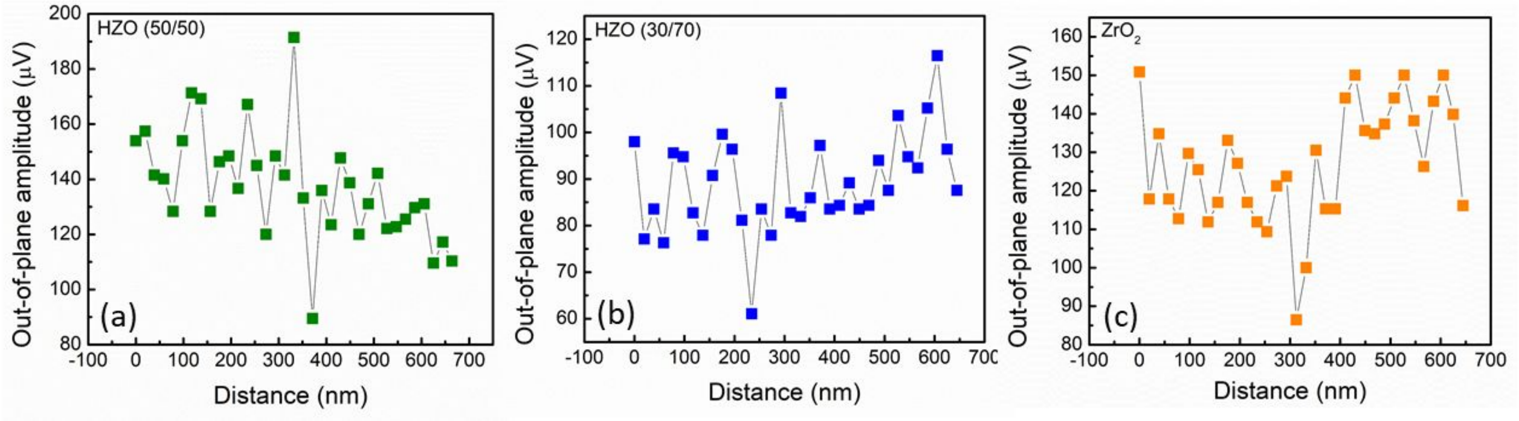

Fig. S2. Out-of-plane amplitude at the boundary of oppositely polarized region marked with black arrows in Fig. 1(g-i) for (a) HZO (50/50), b) HZO (30/70) and (c) ZrO_2, respectively.

Optical properties of the ITO films

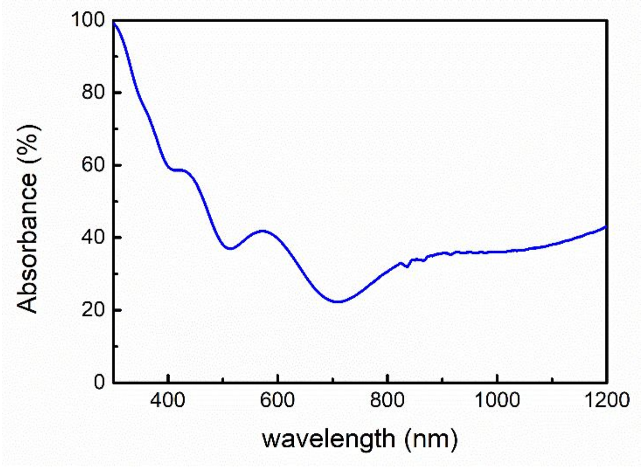

Fig. S3. Absorbance spectrum versus wavelength for an ITO thin film deposited on glass substrate.

Photodetector performance evaluation

The important figure-of-merits determining the photodetection performance of a PD are responsivity (*R*), detectivity (*D**), and sensitivity (*S*), which is evaluated as descrbied in equations S1-S3[5,6]:

$$R = \frac{I_{Light} - I_{Dark}}{P} \tag{S1}$$

$$D^* = \frac{\sqrt{A\Delta f}}{NEP} \tag{S2}$$

$$S = \frac{I_{Light} - I_{Dark}}{I_{Dark}} \tag{S3}$$

where I_{Light}, I_{Dark}, P, A, Δf, and *NEP*, represent the short-circuit current with illumination, short-circuit current without illumination, illumination power, effective area, bandwidth, noise equivalent power, and electron charge, respectively[7]. The dark current (current density) value for the ZrO_2, HZO (50/50) and HZO (30/70)-based PDs are 1.31×10^{-7} A (1.14 μA/cm^2), 2.15×10^{-7} A (1.76 μA/cm^2), and 3.65×10^{-8} A (0.30 μA/cm^2), respectively, while the light current can be extracted from the Figs. 2(a)-(c).

The NEP can be calculated using the equation S4 below[5]:

$$NEP = \frac{RMS}{R} \tag{S4}$$

where *R* is the responsivity of the detector and *RMS* is root mean square of the noise signal. The NEP value for the ZrO_2, HZO (30/70) and HZO (50/70)-based PDs are 2.86×10^{-10},

$1.55x10^{-8}$ and $1.55x10^{-8}$ and $3.24x10^{-7}$ W, respectively. Usually, lower NEP corresponds to a higher sensitivity of the PD, confirming that the Al/Si/SiO_x/ZrO_2/ITO PD is the most sensitive. In addition, the cut-off frequency (f_{-3dB}) was calculated according to the following equation[8]:

$$f_{-3dB} = \left(\frac{0.35}{\tau_r}\right) \quad \text{(S5)}$$

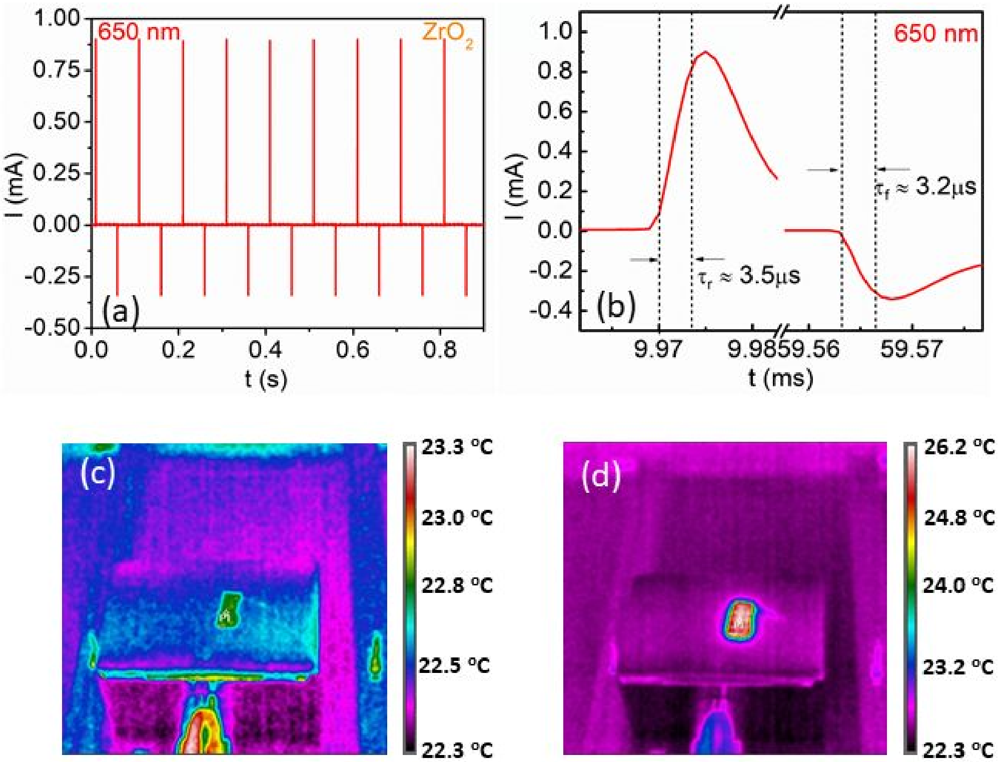

Figure S4. (a) *I–t* curves for the Al/Si/SiO_x/ZrO_2/ITO devices measured for a light of 650 nm wavelength at a fixed chopper frequency of 10 Hz and a fixed power density of 540 mW/cm^2, at 0 V. (b) A typical transient response of the device at 650 nm. Thermal camera images for the Al/Si/SiO_x/ZrO_2/ITO device under (a) dark and (b) under 180 s of light illumination at 650 nm wavelength with a power density 540 mW/cm^2.

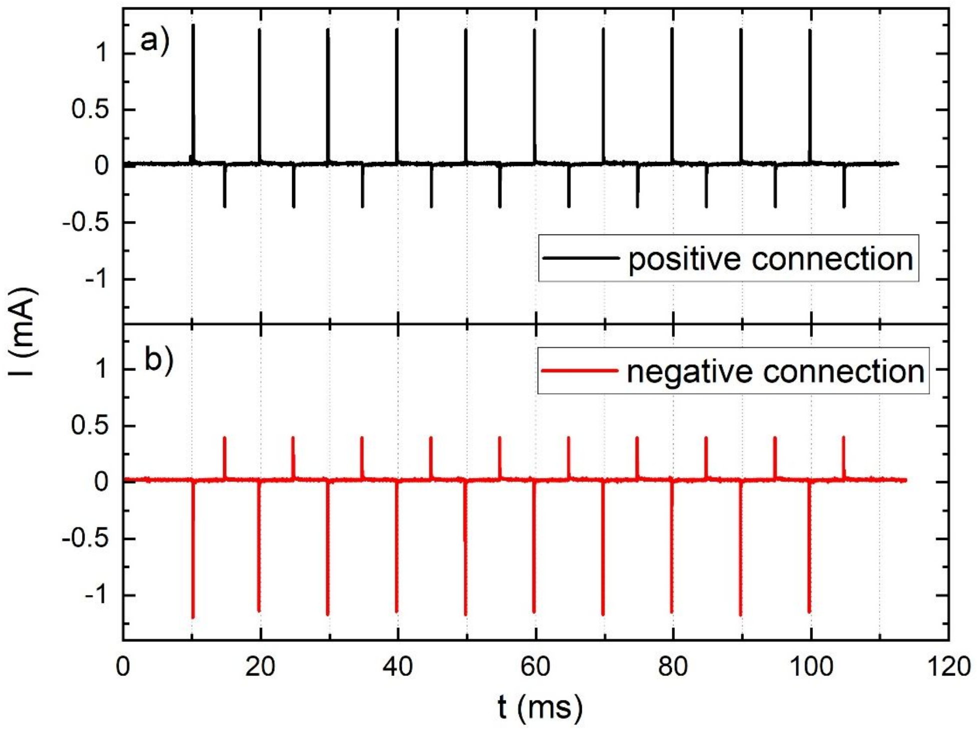

Figure S5. *I*–*t* curves for the $Al/Si/SiO_x/ZrO_2/ITO$ devices measured for a light of 650 nm wavelength at a fixed chopper frequency of 100 Hz and a fixed power density of 251 mW/cm^2, at 0 V, under (a) positive and (b) negative pre-poling conditions.

PPG measurements

Before we undertook the PPG measurements using our device, we tested the photoresponse of a reference Si device without the HZO layer that is present in our device. We observed no response from the sample, but noises as shown in Fig. S5(a). Then, the graphs shown in Fig. S5(b) shows the signal of the Al/Si/SiO_x/ZrO_2/ITO SPD without placing the finger between the light source and the photodetectors, with a red and a near-infrared light of wavelength 650 nm and 940 nm, respectively, at a fixed power density of 1.4 mW/cm^2 and 540 mW/cm^2, and a pulse repetition rate of 10 Hz for both the lights. The measurement was carried out using a laser pulse at 500 Hz for 10 s. Therefore, we observe a constant response. Next, we tested the signal using laser pulses at 500 Hz, which is much higher than the expected heart rate of around 1 Hz, resulting in around 500 samples in every heart beat period available for further analysis. We measured for 10 seconds, resulting in approximately 5,000 peaks total as shown in Fig. S5(c). We then calculated the envelope of these peaks. The idea behind this approach is to maximize the impact of the ferro-pyro-phototronic effect by maximization of the temperature gradient. It is worth noticing, that the only usable part of the signal are the peak value and position, which can be easily recovered using the envelope (inset in Fig. S5(b)).

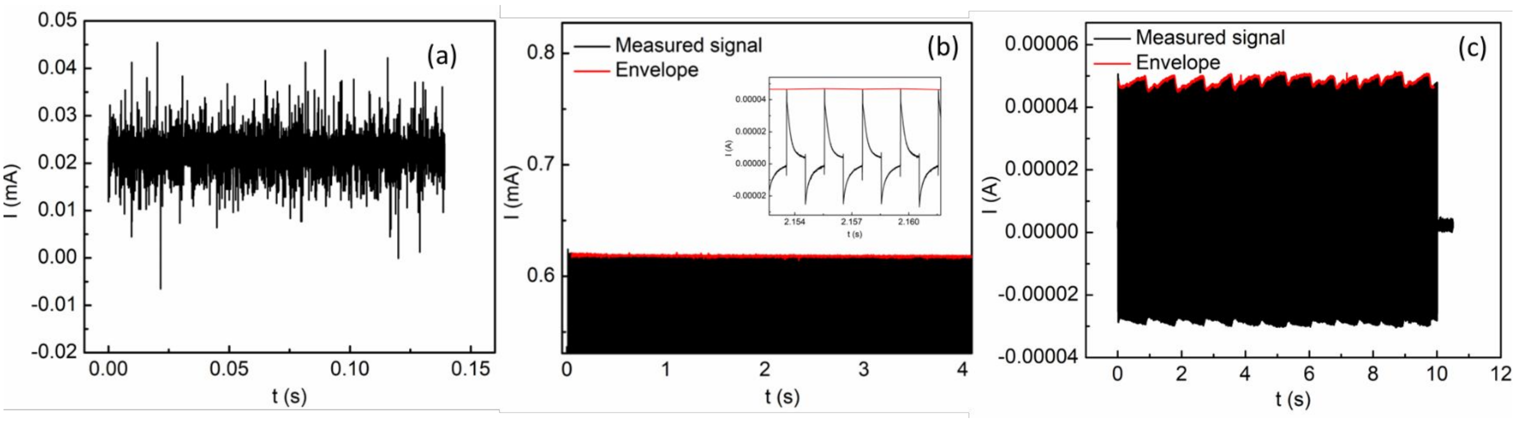

Figure S6. (a) Measured signal without the HZO layer. (b) Reference signal without the finger and the inset figure shows the envelope with red line representing smoothed signal. (c) Signal of heartbeat measured from a finger. The signal in red represents the PPG signal from a Al/Si/SiO_x/ZrO_2/ITO SPD integrated PPG device.

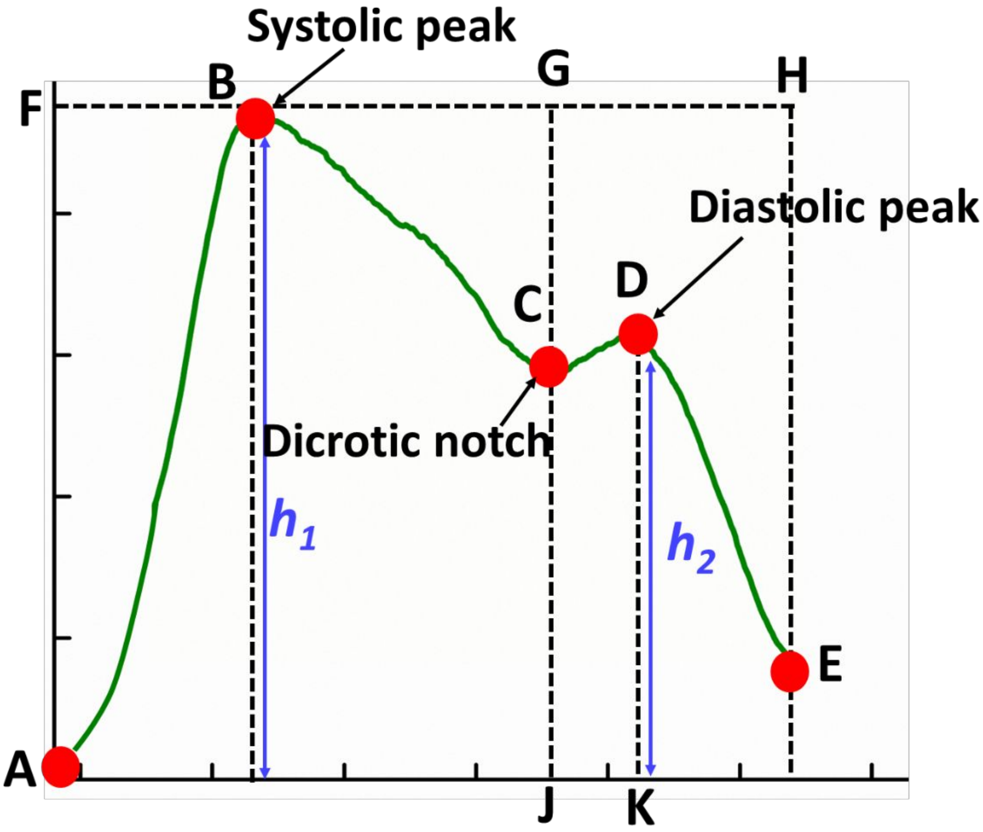

Figure S7. Typical PPG waveform signal for the estimation of the blood pressure.

PPG performance benchmark

Table S2 compares the performance characteristics of the Al/Si/SiO_x/ZrO_2/ITO SPDs of this work as a PPG device with other commercial oximeters and PPG sensors in wristwatches, and also other devices that are currently being reported in the literature. While the information about performance indicators of commercial oximeters and PPG sensors in wristwatches is scarce, it is possible to conclude that our Al/Si/SiO_x/ZrO_2/ITO device is promising since it is self-powered while other devices are not. Also, it shows a slightly higher SNR than the SPOmedical 6106 reflectance finger probe Oximeter which also requires

power. In terms of accuracy, our device shows superior performance when compared to the PPG sensors incorporated in Garmin Venu 2s and Apple Watch Series 7 wristwatches, while it is comparable to the WristOx2 3150 oximeter and Masimo SET® pulse oximeter. It is seen that compared to organic and inorganic PPG devices that are currently being investigated, our device shows improved performance in terms of power consumption/voltage and SNR. Thus, our self-driven Al/Si/SiO_x/ZrO_2/ITO PD is a first step towards the use of CMOS compatible ferroelectric ZrO_2-based SPD as PPG devices in the Healthcare 4.0 applications, where there are clear advantages over current state-of-art or other emerging materials and devices of high sensitivity/accuracy as well as self-powering.

Table S2. Comparison table showing the performance of our device as a PPG device with other PPG devices.

**Device**	**Flexibility**	**Applied voltage (V)**	**Power consumption (mW)**	**Signal-to-noise ratio (dB)**	**Error in SpO_2 estimation (%)**	**Ref.**
Massimo Rad-5[a] Oximeter	Rigid	N/D	520	N/D	N/D	9
Nonin palmSAT 2500[a] Oximeter	Rigid	N/D	195	N/D	N/D	9
SPOmedical 6106 reflectance finger probe Oximeter	Rigid	3.3	3.3	37-39	N/D	9
Masimo SET® pulse Oximeter	Rigid	N/D	N/D	N/D	1.5	10
Garmin Venu 2s wristwatch	Rigid	3.85	ND	N/D	5.8	11
Apple Watch Series 7 wristwatch	Rigid	3.85	N/D	N/D	2.2	11
WristOx2 3150 oximeter	Rigid	3	N/D	N/D	2	12
PEDOT::PSS/TFB/ F8BT/ TBT/ PEN/Al	Flexible	9	0.004	N/D	2	13
ITO/P3HT/ PCBM/ Parylene/Al	Flexible	5	N/D	N/D	N/D	14
Ag/ 3HT/PCBM/Ag	Flexible	0	0	24.4	<2	15
IZO/C_{70}: TAPC/ PET/Al	Flexible	3.3	0.024	N/D	N/D	16
Glass/ITO/C_{60}: DBP/Al	Rigid	3.3	0.008	18	N/D	17
Si photodiode	Rigid	3.3	0.004	29	N/D	18
Si photodiode	Rigid	1.2	0.3	53	<2	19
In-Ga/Si/$Ti_3C_2T_x$/ITO	Rigid	0	0	N/D	N/D	20
FTO/TiO_2/Sb_2Se_3/ITO	Rigid	0	0	N/D	N/D	21
Al/Si/SiO_x/ZrO_2/ITO	Rigid	0	0	41	<2	**This work**

TFB- poly(9,9-dioctylfluorene-co-n-(4-butylphenyl)-diphenylamine), F8BT- poly((9,9-dioctylfluorene-2,7-diyl)-alt-(2,1,3-benzothiadiazole-4, 8-diyl)), TBT- poly((9,9-dioctylfluorene-2,7-diyl)-alt-(4,7-bis(3-hexylthiophene-5-yl)-2,1,3-benzothiadiazole)-20,20-diyl), PEN-polyethylene naphthalate, PCBM- poly(3-hexylthiophene) :(6,6)-phenyl-C61-butyric acid methyl ester, TAPC-4,4′-cyclohexylidenebis[N,N-bis(4- methylphenyl)benzenamine], PET- polyethylene

terephthalate, DBP-Dibutyl phthalate, PEDOT:PSS-Poly(3,4-ethylenedioxythiophene) polystyrene sulfonate, P3HT-Poly(3-hexylthiophene), FTO-Fluorine Tin oxide.

References

1. Hyuk Park, M., Joon Kim, H., Jin Kim, Y., Lee, W., Moon, T. and Seong Hwang, C. Evolution of phases and ferroelectric properties of thin $Hf_{0.5}Zr_{0.5}O_2$ films according to the thickness and annealing temperature. *Appl. Phys. Lett.* **102**, 242905 (2013).
2. Kashir, A., Kim, H., Oh, S. and Hwang, H., Large remnant polarization in a wake-up free $Hf_{0.5}Zr_{0.5}O_2$ ferroelectric film through bulk and interface engineering. *ACS Appl. Electron. Mater.* **3**, 629-638 (2021).
3. Hsain, H.A., Lee, Y., Parsons, G. and Jones, J.L. Compositional dependence of crystallization temperatures and phase evolution in hafnia-zirconia $(Hf_xZr_{1-x})O_2$ thin films. *Appl. Phys. Lett.* **116**, 192901 (2020).
4. Xu, B., Lomenzo, P.D., Kersch, A., Schenk, T., Richter, C., Fancher, C.M., Starschich, S., Berg, F., Reinig, P., Holsgrove, K.M. and Kiguchi, T. Strain as a global factor in stabilizing the ferroelectric properties of ZrO_2. *Adv. Funct. Mater.* **34**, 2311825 (2024).
5. Silva, N. E., Jayakrishnan, A. R., Kaim, A., Gwozdz, K., Domingues, L., Kim, J. S., Istrate, M. C., Ghica, C., Pereira, M., Marques, L., Gomes, M. J. M., Hoye, R. L. Z., MacManus-Driscoll, J. L., Silva, J. P. B. *Adv. Funct. Mater.* **35**, 2416979 (2025).
6. Silva, J.P., Vieira, E.M., Gwozdz, K., Silva, N.E., Kaim, A., Istrate, M.C., Ghica, C., Correia, J.H., Pereira, M., Marques, L. and MacManus-Driscoll, J.L. High-performance and self-powered visible light photodetector using multiple coupled synergetic effects. *Mater. Horiz.* **11**, 803-812 (2024).
7. Peng, J., Jiang, J., Yuan, S., Hou, P. and Wang, J., Harnessing the power of temperature gradient-enhanced pyroelectricity: Self-powered temperature/light detection in Ce-doped HfO_2 ferroelectric films with downward spontaneous polarization. *J Materiomics.* **11**, 100911 (2025).
8. Yu, B., Deng, Y., Luo, Z., Wang, M., Zhuang, Y., Chen, X., Liu, S. and Zhao, Q. Platinum (II)-acetylide conjugated polymer containing aza-BODIPY moieties for panchromatic photodetectors. *Adv. Electron. Mater.* **8**, 2101323 (2022).

9. Glaros, K.N. and Drakakis, E.M. A sub-mW fully-integrated pulse oximeter front-end. *TBioCAS*. **7**, 363-375 (2012).
10. https://professional.masimo.com/products/sensors/rd-set/.
11. Jiang, Y., Spies, C., Magin, J., Bhosai, S.J., Snyder, L. and Dunn, J. Investigating the accuracy of blood oxygen saturation measurements in common consumer smartwatches. *PLOS Digital Health*. **2**, e0000296 (2023).
12. https://mhealth.jmir.org/2019/6/e12866/
13. Lochner, C.M., Khan, Y., Pierre, A. and Arias, A.C. All-organic optoelectronic sensor for pulse oximetry. *Nat. Commun*. 5, 5745 (2014).
14. Yokota, T., Zalar, P., Kaltenbrunner, M., Jinno, H., Matsuhisa, N., Kitanosako, H., Tachibana, Y., Yukita, W., Koizumi, M. and Someya, T. Ultraflexible organic photonic skin. *Sci. Adv*. 2, e1501856 (2016).
15. Ganser, R., Bongarz, S., von Mach, A., Azevedo Antunes, L. and Kersch, A. Piezo-and pyroelectricity in zirconia: A study with machine-learned force fields. *Phys. Rev. Appl.* **18**, 054066 (2022).
16. Lee, H., Kim, E., Lee, Y., Kim, H., Lee, J., Kim, M., Yoo, H.J. and Yoo, S. Toward all-day wearable health monitoring: An ultralow-power, reflective organic pulse oximetry sensing patch. *Sci. Adv*. **4**, eaas9530 (2018).
17. Elsamnah, F., Bilgaiyan, A., Affiq, M., Shim, C.H., Ishidai, H. and Hattori, R. Reflectance-based organic pulse meter sensor for wireless monitoring of photoplethysmogram signal. *Biosensors*. **9**, 87 (2019).
18. Caizzone, A., Boukhayma, A. and Enz, C., 2019. A 2.6μW monolithic CMOS photoplethysmographic (PPG) sensor operating With 2 μW LED power for continuous health monitoring. *TBioCAS*. **13**, 1243-1253 (2019).
19. Mazandarani, M.S., Bostani, R., Papi, R., Ebrahimi, Z., Koleibi, E.R., Fontaine, R., Gagnon-Turcotte, G. and Gosselin, B., A Highly Duty-Cycled PPG Sensor With Ultra-Low-Power Consumption and Wide Input Range. *IEEE Sens. J.* **24**, 39169-39181 (2024).
20. Song, L., Xu, E., Yu, Y., Jie, J., Xia, Y., Chen, S., Jiang, Y., Xu, G., Li, D. and Jie, J. High-barrier-height $Ti_3C_2T_x$/Si microstructure Schottky junction-based self-powered photodetectors for photoplethysmographic monitoring. *Adv. Mater. Technol*. **7**, 2200555 (2023).

21. Gao, S., Xu, J., Shi, S., Chen, J., Xu, J., Kong, L., Zhang, X. and Li, L., High-performance Self-powered Ultraviolet-visible-near infrared Broadband Photodetectors Based on Sb_2Se_3/TiO_2 Heterojunctions and Multifunctional Applications. *Adv. Opt. Mater.* **12**, 2302141 (2024).
